# Investigation on the Interfaces in Organic Devices by Photoemission Spectroscopy

**DOI:** 10.3390/nano15090680

**Published:** 2025-04-30

**Authors:** Haipeng Xie, Xianjun Cheng, Han Huang

**Affiliations:** Hunan Key Laboratory of Super-Microstructure and Ultrafast Process, School of Physics, Central South University, Changsha 410083, China; xiehaipeng@csu.edu.cn (H.X.); chengxianjun@csu.edu.cn (X.C.)

**Keywords:** organic semiconductors, band bending, interface engineering, photoelectron spectroscopy

## Abstract

Organic semiconductors have garnered significant interest owing to their low cost, flexibility, and suitability for large-area electronics, making them vital for burgeoning fields such as flexible electronics, wearable devices, and green energy technologies. The performance of organic electronic devices is crucially determined by their interfacial electronic structure. Specifically, interfacial phenomena such as band bending significantly influence carrier injection, transport, and recombination, making their control paramount for enhancing device performance. This review investigates the interplay among molecular orientation, interfacial charge transfer, and interfacial chemical reactions as the primary drivers of interface band bending. Furthermore, it critically examines effective strategies for optimizing interfacial properties via interface engineering, focusing on interlayer insertion and template layer methods. The review concludes with a summary and outlook, emphasizing the integration of interface design with material development and device architecture to realize next-generation, high-performance organic electronic devices exhibiting improved efficiency and stability.

## 1. Introduction

Organic semiconductors have attracted significant attention because of their low cost, flexibility, and suitability for large-area electronics. These features are playing an increasingly important role in the flexible electronics, wearable devices, and green energy technologies [1,2,3,4]. Applications include organic light-emitting diodes (OLEDs), organic photovoltaics (OPVs), and organic field-effect transistors (OFETs) [5,6,7,8,9,10,11,12,13]. However, organic semiconductor device performance is often limited by complex interfacial phenomena. Understanding and controlling these interfaces requires characterizing their electronic structure. This structure includes energy level alignment, interface dipoles, and band bending. Moreover, the interactions at these interfaces, such as charge transfer or chemical reactions, are often complex and depend on the materials involved. For instance, interfaces like In/CuPc can be non-reactive, whereas others, such as C_60_F_18_ on Ni(100), exhibit strong chemical interactions [14,15]. The nature and strength of these interactions directly influence interfacial electronic phenomena, with band bending being particularly significant. Band bending reflects the electrostatic potential distribution near the interface, critically affecting carrier behavior, including injection barriers and transport pathways. Wang et al. fabricated a F_16_CuPc/BP2T organic heterojunction and utilized interface band bending in this device [16]. The band bending effectively promoted electron and hole accumulation in the respective material layers. This approach successfully created bipolar OFETs, which exhibited high mobility and balanced transport characteristics. Similarly, Chen et al. utilized organic semiconductor heterojunctions as charge generation layers to enhance the performance of tandem OLEDs [17]. Oehzelt et al. provided a crucial theoretical framework, which combines an electrostatic model with the density of states distribution of organic semiconductors [18]. It helps elucidate energy level alignment at metal/organic and organic/organic interfaces and explains the origin of band bending. This framework is crucial for guiding high-performance device design.

**Figure 1:**
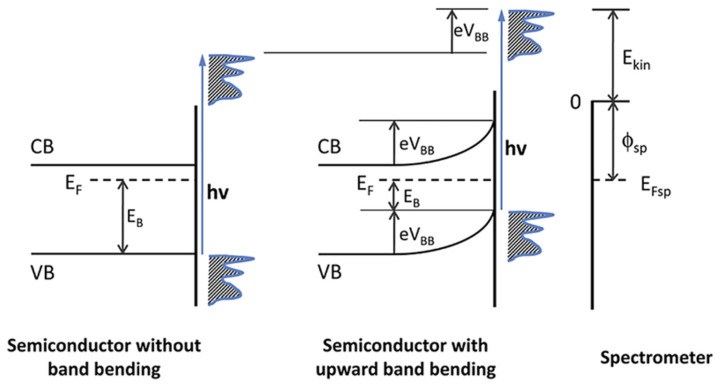
Energy diagram of the photoelectric emission process [22].

Understanding interfacial phenomena is crucial for device performance and requires powerful surface-sensitive characterization techniques. Among these, photoelectron spectroscopy (PES), particularly ultraviolet (UPS) and X-ray (XPS) photoelectron spectroscopy, is a core tool for revealing organic interfaces. The basic principle of PES involves irradiating a sample with photons (hν) and measuring the kinetic energy of the emitted photoelectrons. This kinetic energy directly relates to the electrons’ original binding energy in the material. Analyzing relevant spectral regions (UPS valence band/cutoff, XPS core levels) yields crucial information about interfacial electronic structure. Band bending specifically involves an overall shift of electronic energy levels near the semiconductor surface relative to the bulk or substrate (Figure 1). While a core level’s binding energy relative to the local Fermi level may remain constant, its energy position relative to a fixed external reference changes. Therefore, monitoring the apparent binding energy (or kinetic energy) shift of characteristic core levels before and after interface formation directly quantifies the magnitude and direction of band bending. Indeed, the widespread application of PES across diverse material systems has provided an in-depth understanding of organic semiconductor interfacial electronic structure [19,20,21].

Interface engineering provides an effective way to modulate energy level alignment and band bending, improving organic semiconductor device performance. Researchers have focused on precisely controlling interface properties using interlayer materials. Zhang et al. [23] introduced a high-work-function PEDOT:PSS buffer layer into a pentacene system, significantly improving device conductivity. Zhu et al. used a molybdenum trioxide (MoO_3_) buffer layer to adjust energy level alignment at the C8-BTBT/cobalt (Co) interface [23]. They revealed how MoO_3_ effectively modulates interface band bending, reduces the hole injection barrier, and induces Fermi level pinning. Chen et al. reduced the charge injection barrier by introducing a MoO_3_ buffer layer in a C_60_/pentacene system. C-V testing showed that the MoO_3_ buffer layer decreased the turn-on voltage and increased capacitance, promoting charge injection. This resulted in a maximum device power efficiency of 21.9 lm/W [17]. In OLEDs, band bending at the electrode/organic interface affects the balance of electron and hole injection and transport, which is crucial for maximizing light emission efficiency. Using a graphene anode and a MoO_3_ interlayer can induce band bending in OLEDs, optimizing charge transport energy level alignment and achieving higher power efficiency than traditional ITO devices. These studies demonstrate the crucial role of interface engineering in optimizing device performance and enhancing reliability. Besides interlayers, template layers are also important for improving organic semiconductor device performance. Qian et al. fabricated a copper phthalocyanine (CuPc) thin film on a p-6P template layer [24], creating a high-performance organic heterojunction phototransistor with a responsivity of 4.3 × 10^2^ A/W. Recent studies have further explored the p-6P template layer’s role in CuPc growth [25]. For instance, using four aromatic molecules similar to p-6P as template layers, researchers found that fluorinated p-6P (p-6PF) can enhance the electron field-effect mobility of F_10_-SiPc OTFTs. Furthermore, optimizing substrate surface inertness to control the p-6P template layer nanostructure significantly improves CuPc transistor performance [26].

**Figure 2:**
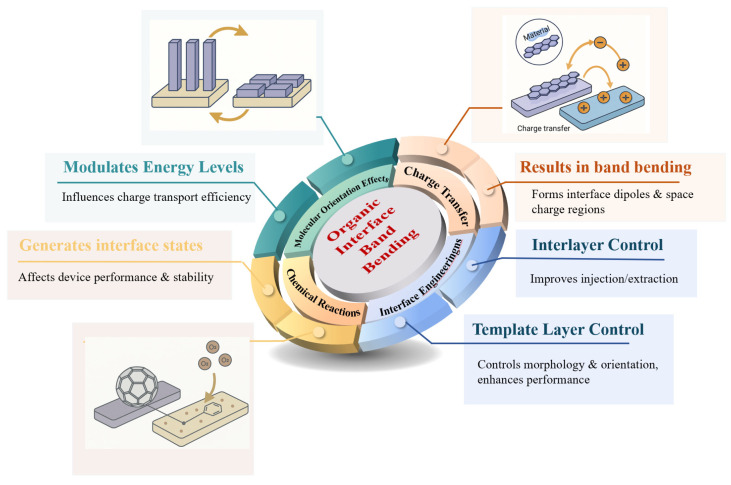
Comprehensive schematic of organic interface band bending and its impact on device performance.

This review examines how three key factors regulate electronic structure at organic semiconductor interfaces. These factors are molecular orientation, interfacial charge transfer, and interfacial chemical reactions. The discussed electronic structure includes energy level alignment, interface dipoles, and band bending. The review discusses interface engineering strategies. These strategies optimize interface properties. Particular focus is placed on interlayer insertion and template layer methods, as schematically summarized in Figure 2. Finally, the review summarizes research in this field. It also provides an outlook on future directions.

## 2. Substrate-Controlled Growth of Organic Molecular Thin Films

The physical structure of organic semiconductor thin films, encompassing their growth characteristics and molecular arrangement, is fundamental to the performance of electronic devices built from them. Controlling this structure requires a detailed understanding of the film formation process on various substrates. This section explores key aspects of substrate-controlled growth. Specific topics covered include the discussion of different thin film growth modes, the influence of critical deposition parameters such as substrate temperature and deposition rate, the significance of molecular orientation, and common strategies employed to control this orientation.

### 2.1. Growth of Organic Molecules

#### 2.1.1. Organic Molecular Growth Modes

**Figure 3:**
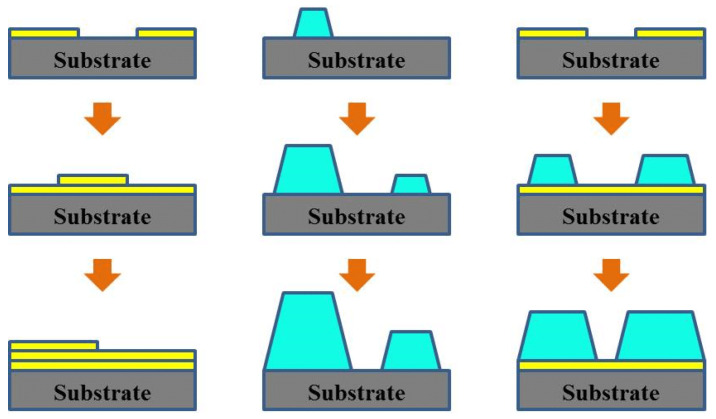
Three Thin Film Growth Modes. Illustrates the three modes of organic thin film growth on a substrate: layer-by-layer growth (Frank–van der Merwe), island growth (Volmer–Weber), and layer-plus-island growth (Stranski–Krastanov) [37].

The microstructure of organic semiconductor thin films, particularly molecular growth mode and orientation, crucially determines device performance. Figure 3 illustrates the three main modes of organic thin film growth on a substrate. Generally, the two-dimensional layer-by-layer growth mode (Frank–van der Merwe type) promotes high carrier mobility in semiconductor thin films [27,28,29]. Using scanning tunneling microscopy (STM), Tsuchie et al. studied C_60_ grown on the Si(111)-$\sqrt{3}\times \sqrt{3}$-Ag surface. They observed a hexagonal close-packed structure with a layer thickness of approximately 0.95 nm, which matches the C_60_ molecular diameter [30]. This layer-by-layer growth likely occurs partly because the periodic potential field from the Ag surface reconstruction matches the C_60_ molecule size. This match allows intermolecular interactions to dominate the growth process. Experiments show that using a layer-by-layer grown C_60_ thin film as an electron transport layer in OFETs can result in a switching ratio as high as 10^6^ and a carrier mobility of 0.1 cm^2^/Vs [31]. Jeong et al. synthesized a hexathiophene derivative with symmetric hydroxypropyl groups. These groups suppressed three-dimensional island growth via interlayer and lateral intermolecular hydrogen bonding, leading to nearly ideal two-dimensional layer-by-layer crystallization [32]. In contrast, the three-dimensional island growth mode (Volmer–Weber type) can create voids at film boundaries and generate defects like grain boundaries, significantly hindering charge transport [29,33,34,35]. However, most organic molecules follow the Stranski–Krastanov growth mode. While initial deposition might show some layer-by-layer growth, the growth typically transitions to forming three-dimensional islands after the first monolayer covers the substrate. This transition forces charge carriers to bypass laterally stacked molecular islands, increasing the transport path and reducing efficiency. For example, C8-BTBT growth on Au follows a typical layer-plus-island mode. Atomic force microscopy (AFM) shows that, in the initial 1.8 nm thick layer, C8-BTBT molecules lie down (long axis parallel to the surface) [36]. As the film thickness increases, accumulating interfacial stress causes the molecules to adopt an upright orientation. This transition eventually leads to the formation of island structures approximately 2.7 nm high. This gradient in molecular orientation leads to interface band bending: the vacuum level (E_vac_) shifts down by 0.76 eV, and the highest occupied molecular orbital (HOMO) level shifts up by 0.18 eV, decreasing the hole injection barrier to 1.72 eV.

#### 2.1.2. Temperature and Rate Control of Organic Molecular Growth

The growth of organic semiconductor thin films is influenced by several parameters, notably substrate temperature (T_sub_) and deposition rate. These factors are crucial for controlling the film microstructure, morphology, and ultimate device performance. They collectively govern behaviors such as adsorption, diffusion, nucleation, and molecular growth on the substrate surface.

The T_sub_ primarily influences the surface mobility of molecules. Increasing T_sub_ provides adsorbed molecules with enough thermal energy to overcome diffusion and rearrangement barriers. This enhanced mobility allows them to diffuse further and provides more time to relax into energetically favorable sites [38,39,40,41,42]. This favors the formation of larger, more ordered domains with fewer defects. For example, consider pentacene thin films grown on SiO_2_ or OTS-treated SiO_2_ substrates [43]. Increasing T_sub_ from room temperature to 80 °C significantly increases island grain size. Grain sizes expand from tens to hundreds of nanometers. Studies of pentacene transistors fabricated on OTS-SiO_2_ show that carrier mobility increases as T_sub_ increases from 25 °C, reaching a peak at approximately 60 °C (1.12 cm^2^/Vs). However, the impact of temperature is not always beneficial. Excessively high temperatures can trigger phase transitions in the material. For example, pentacene transitions from a thin film phase to a bulk phase at approximately 207 °C [44,45]. PbPc provides another example. It transforms into a triclinic phase when T_sub_ exceeds 140 °C [46,47]. Phase transitions alter molecular packing and grain boundary structure. Consequently, device performance shows a non-linear temperature dependence and may even decline at higher temperatures.

The deposition rate controls the flux of molecules arriving at the substrate per unit time. A lower deposition rate allows molecules more time for diffusion and relaxation on the surface, bringing the growth process closer to thermodynamic equilibrium. This condition generally favors the formation of films with larger size, higher crystallinity, and high phase purity. Conversely, a higher deposition rate shortens the effective diffusion time for molecules, leading to a significant increase in nucleation density [48,49,50]. For example, during pentacene growth on SiO_2_, increasing the deposition rate from 0.06 to 0.6 nm/min raises the island density nearly tenfold [48]. This often leads to smaller grains and higher grain boundary density. At high deposition rates, the growth process becomes kinetically limited. This can lead to increased structural disorder, the formation of amorphous phases, or the trapping of energetically supersaturated metastable structures within the film. These metastable structures may relax during subsequent processing or device operation, impacting the long-term stability of the device. However, the relationship between growth rate and performance is complex. Yogev et al. studied pentacene thin films using conductive atomic force microscopy and found that, despite a reduction in grain size with increasing deposition rate, device mobility improved [51]. This was attributed to the relatively enhanced conductivity of the grain boundaries at high deposition rates, which lowers the potential barrier for charge transport across these boundaries.

Obtaining optimal film morphology and device performance typically requires finding a balance between the deposition rate and T_sub_. Slow deposition allows ample time for diffusion and ordered arrangement, but a suitable T_sub_ is necessary to provide sufficient diffusion kinetics. Overall, decreasing the deposition rate enhances the crystalline quality of thin films, consequently enhancing carrier mobility. For instance, pure pentacene deposited by organic molecular beam epitaxy (OMBE) at 0.5 Å/s yielded a mobility of 0.5 cm^2^/Vs; lowering the deposition rate to 0.2 Å/s increased the mobility to 0.62 cm^2^/Vs [52,53]. Furthermore, pentacene single crystals grown by physical vapor transport (PVT) at extremely slow rates (≤5 × 10^−7^ cm/s) have enabled the fabrication of OFET devices with hole mobilities reaching approximately 8 cm^2^/Vs [54]. These examples underscore that reducing the growth rate under suitable temperature conditions is crucial for obtaining high-quality crystalline films and achieving high-mobility devices.

### 2.2. Organic Molecular Orientation

#### 2.2.1. Influence of Molecular Orientation on Charge Transport and Interface Energy Levels

**Figure 4:**
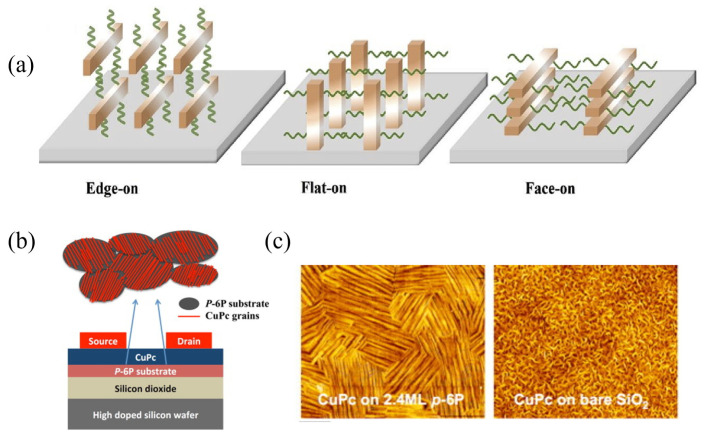
Effect of Organic Molecule Orientation on Charge Transport. (**a**) Schematic illustration of three different orientations of organic molecules on a substrate: Edge-on, Flat-on, and Face-on, which affect the efficiency of charge transport [66]. (**b**) Schematic diagram of CuPc film grains on a p-6P substrate and the device structure of a bottom-contact CuPc thin film transistor. (**c**) AFM images of CuPc thin films on different substrates [65].

Besides growth mode, molecular orientation is another important interfacial property influencing device performance. It significantly affects light absorption and charge transport within thin films. For conjugated molecules, orientation is typically classified into three main types (Figure 4a): edge-on, flat-on, and face-on. In the edge-on orientation, the molecular conjugated plane is roughly perpendicular to the substrate surface. The π–π stacking direction is parallel to the substrate plane. This arrangement allows effective intermolecular π-orbital overlap within the substrate plane. This greatly promotes in-plane charge transport within the film. Therefore, the edge-on orientation is highly advantageous for devices requiring high in-plane charge mobility. In OFETs, current flows between the source and drain electrodes primarily in the channel parallel to the substrate [55,56,57]. The edge-on orientation aligns the efficient π–π stacking transport pathways with this current direction. For example, materials like triisopropylsilylethynyl pentacene (TIPS-PEN) often adopt this orientation to achieve high mobility [58]. In contrast, the face-on orientation has the molecular conjugated plane roughly parallel to the substrate surface. Here, the π–π stacking direction is perpendicular to the substrate plane. This configuration favors charge transport perpendicular to the substrate (out-of-plane). Devices requiring vertical charge transport include OPVs, OLEDs, and diodes. For these devices, the face-on orientation is generally more ideal. This orientation aligns the efficient π–π stacking transport pathways with the vertical current direction.

More importantly, molecular orientation directly controls the interfacial electronic structure. Molecular orientation determines which part of the molecule is exposed to vacuum or contacts an adjacent layer. Consequently, the effective work function (WF), ionization potential (IP), and electron affinity (EA) of the film surface show orientation dependence. Different orientations can generate different surface dipoles. These dipoles alter the vacuum level. Therefore, controlling molecular orientation is an effective way to tune interface properties, including energy level alignment, interface dipoles, and charge injection/extraction barriers. This topic is discussed in detail in Section 3.1.1.

#### 2.2.2. Control of Molecular Orientation

Researchers have developed various strategies to effectively control molecular orientation. These methods fall mainly into two categories: molecular design, achieved by adjusting the molecular structure, and physical methods that utilize external conditions. Physical methods include controlling thermodynamics or combining the solution state with film formation kinetics [59,60,61,62]. While both approaches are employed, this section focuses on physical methods, particularly the widely used technique of template layer induction. Template layer induction is a common and effective technique. This strategy involves pre-constructing a highly ordered, ultrathin organic film on a substrate; it is particularly effective on amorphous substrates. This film serves as an inducing layer, often called a template layer. It effectively replaces the original substrate surface. It provides a regular template with specific structural information that guides subsequent growth. Its core advantage lies in enabling the fabrication of high-quality, highly oriented organic semiconductor films. This technique is particularly useful on substrates where achieving direct ordered growth is difficult. Keitaro et al. used π–π interactions between an rGO template layer and CuPc molecules [63]. These interactions effectively induced a face-on orientation for the CuPc molecules (molecular plane parallel to the substrate). This orientation aligns the intermolecular π–π stacking direction with the charge transport direction perpendicular to the substrate. This provides a more efficient transport path for charge carriers. Ultimately, it significantly increases vertical carrier mobility. Schünemann et al. studied molecules like zinc phthalocyanine (ZnPc) and diindeno[1,2,3-cd:1′,2′,3′-lm]perylene (DIP) [64]. They deposited these on a strongly interacting metal underlayer and a PTCDA template layer. They found the molecular orientation in the resulting crystalline films could be tuned from nearly upright to more inclined. Gu et al. used ultrathin p-6P as a template layer to induce CuPc molecules to form highly ordered thin films (Figure 4b,c) [65]. This approach significantly improved the carrier mobility in the resulting organic thin film transistors (OFETs).

## 3. Interfacial Electronic Structure and Modulation

Building upon the previous discussion of organic thin film microstructure formation, this section focuses on the resulting interfacial properties. These properties fundamentally control charge carrier dynamics at the interface and therefore play a crucial role in overall device performance. This section examines the influence of three key factors on organic semiconductor interfacial properties: molecular orientation, interfacial charge transfer, and interfacial chemical reactions. Understanding these factors provides theoretical guidance for interface engineering aimed at developing high-performance organic electronic devices.

### 3.1. Mechanisms of Interface Band Bending

#### 3.1.1. Interface Band Bending Induced by Molecular Orientation

As mentioned above, molecular orientation is a critical factor in determining the electronic properties of organic semiconductors, particularly in π-conjugated systems. Distinct molecular orientations (e.g., face-on and edge-on configurations) directly modulate charge transport efficiency by controlling the extent of molecular orbital overlap with adjacent layers or electrodes. This variation in overlap directly influences the efficiency of charge transport pathways and subsequently affects the energy level alignment at the interface. Therefore, molecular orientation is an important parameter for tuning interface energy levels, enabling control over energy barriers and charge injection efficiency in organic devices [21,67].

Duhm et al. found that flat-lying 6T and DH6T films exhibit increased ionization potentials, attributed to a surface dipole layer formed by the ordered molecular arrangement [68]. They suggested that the dipole arises from the intramolecular dipole moment between the negatively charged π electron cloud and the positively charged molecular plane. In contrast, upright molecular layers lack such a dipole structure. Studies on metal phthalocyanine (MPc) molecules on methylammonium lead iodide (MAPbI_3_) surfaces show that face-on MPc molecules have stronger adsorption energy and facilitate hole transfer, unlike the edge-on configuration, which presents a larger energy barrier [69]. These findings highlight how molecular orientation affects interfacial charge transfer and energy level modulation.

**Figure 5:**
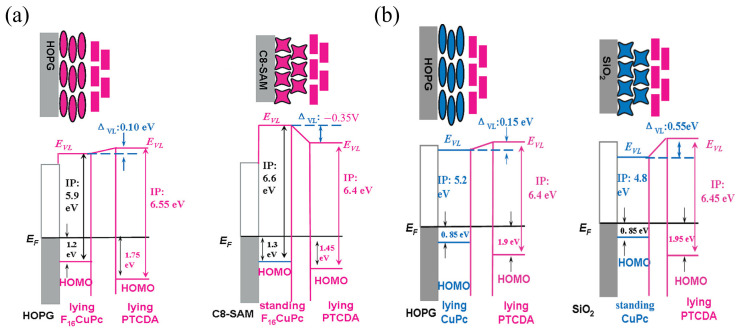
Influence of Molecular Orientation on Energy Level Alignment at Different Interfaces. (**a**) shows energy level diagrams for F_16_CuPc and PTCDA molecular layers on HOPG and C8-SAM. (**b**) shows energy level diagrams for CuPc and PTCDA molecular layers on HOPG and SiO_2_ [77].

Recent studies show that ionization potentials (IPs) of organic thin films are orientation-dependent [68,70,71,72,73,74,75]. IP is the energy difference between the HOMO level and the E_vac_, analogous to how metal work function depends on crystal surface orientation. The α,ω-dihexyl-sexithiophene (DH6T) and α-sexithiophene (6T) thin films on Ag(111) show a significant IP difference of up to 0.6 eV, closely related to the molecules’ flat-lying or upright orientation on the substrate [76,77]. For the planar conjugated molecule CuPc, molecular orientation significantly impacts interfacial electronic structure and energy level alignment. Studies show that the IP of upright (edge-on) thin films is 4.75 eV, while that of flat-lying (face-on) thin films is 5.15 eV. This indicates a higher HOMO level for flat-lying CuPc, attributed to opposing C-H dipole bonds within the molecule. Furthermore, molecular fluorination can alter the built-in surface dipole polarity in the molecular film. Because fluorine is more electronegative than carbon, fluorination can reverse the orientation dependence of the organic thin film’s IP [78]. As illustrated in Figure 5, studies investigated flat-lying 3,4,9,10-perylenetetracarboxylic dianhydride (PTCDA) thin films deposited on both standing (edge-on) and lying-down (face-on) fluorinated copper phthalocyanine F_16_CuPc or CuPc films. These studies showed that the orientation-dependent IP of the underlying F_16_CuPc or CuPc Xfilm strongly influences the formation of the interface dipole at the heterojunction [77]. The C 1s core level and the HOMO of PTCDA on flat-lying F_16_CuPc thin films are 0.3 eV higher than on standing F_16_CuPc thin films, indicating that selecting appropriate molecular orientations can control energy level alignment at organic–organic interfaces [79].

**Figure 6:**
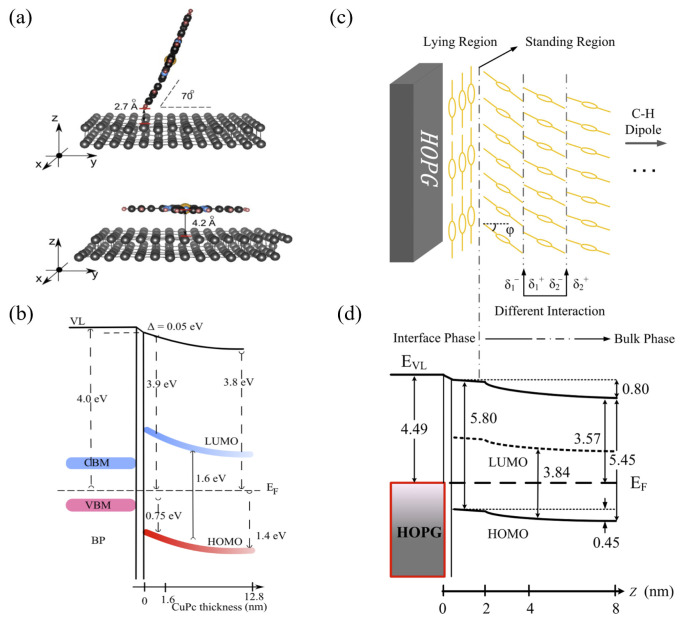
Molecular Arrangement and Energy Level Alignment of CuPc on BP and HOPG. (**a**) Two adsorption configurations of CuPc on BP: flat-lying and upright. (**b**) Energy level diagram of the CuPc/BP interface as a function of CuPc thickness [80]. (**c**) Schematic of CuPc molecular packing on HOPG, showing flat-lying and upright regions. (**d**) Energy level alignment of interfacial and bulk phases of CuPc on HOPG [81].

Layered two-dimensional (2D) semiconductors often possess unique electronic structures and tunable band properties, making them ideal platforms for studying interfacial phenomena such as charge transfer. Black phosphorus (BP) is one such material that has garnered significant interest in these studies. As shown in Figure 6a,b, Wang et al. investigated the impact of CuPc molecular orientation on interface energy level bending in CuPc/BP heterojunctions [80]. The growth orientation of CuPc molecules significantly affects the evolution of the interface electronic structure, thereby modulating interfacial energy level alignment. At low coverage (<1.6 nm), CuPc molecules lie flat on the BP substrate, interacting via weak van der Waals forces. This results in a HOMO energy level close to the Fermi level (initial edge at 0.75 eV) and the IP is 4.50 eV. Conversely, at high coverage (>1.6 nm), increased intermolecular interactions force CuPc into a standing orientation. The weakened π–π stacking and rearranged intramolecular dipoles cause a substantial 0.7 eV shift of the HOMO level towards higher binding energy, increasing the IP to 5.00 eV. These findings highlight molecular orientation as a critical factor in controlling the electronic structure. Further analysis revealed the interplay of surface dipoles and band bending in modulating interface energy levels. The C-H bond dipoles in flat-lying CuPc create a minor interface dipole (0.05 eV), which slightly shifts the vacuum level E_vac_. The enhanced edge polarization of standing CuPc further influences the interface dipole distribution. Thus, the interface dipole is a key regulator of interface energy level bending, and it can be effectively controlled by altering the molecular orientation.

Similarly, Lyu and colleagues investigated the interface electronic structure between organic semiconductor C8-BTBT and HOPG substrate, further substantiating the influence of molecular orientation on interface energy level alignment [23]. As shown in Figure 6c,d, C8-BTBT molecules tend to adsorb in a flat-lying orientation on the HOPG surface in the initial deposited layer. As the film thickness increases beyond 2 nm, the molecular orientation gradually transitions to an upright configuration. Concurrently, the IP decreases from 5.8 eV in the initial thin film (2 nm) to 5.45 eV in the thicker film (8 nm), representing a reduction of 0.35 eV. Spectroscopic analyses provided deeper insights, demonstrating that the HOMO energy level, the S 2p and C 1s core levels, and the E_vac_ all shift downward synchronously as the film thickens. This total shift ranges from 0.4 eV to 0.8 eV. The findings unambiguously identify the change in molecular orientation as the primary mechanism driving energy level bending. In early film growth, when the flat-lying configuration prevails, the non-polar π-conjugated core is more prominently exposed on the film surface, resulting in a relatively weak surface dipole. As the film thickens and molecules reorient upright, the hydrophobic alkyl chains containing C-H polar bonds become increasingly exposed, creating a stronger surface dipole.

#### 3.1.2. Interface Band Bending Arising from Interfacial Charge Transfer

**Figure 7:**
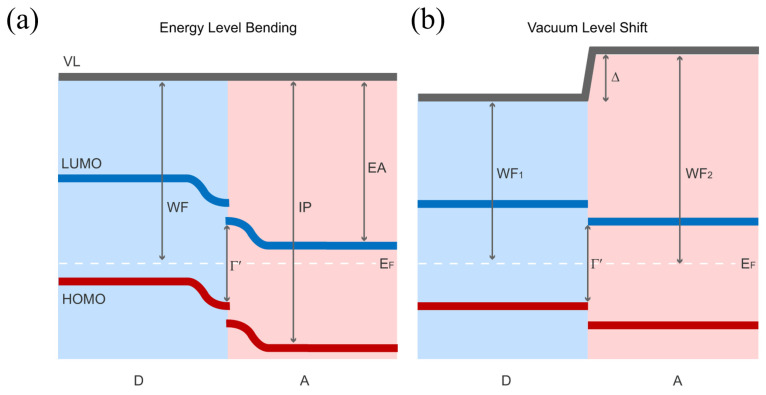
Energy Level Alignment at Organic Donor–Acceptor Interfaces. (**a**) Schematic illustration of energy level bending after the donor and acceptor are in contact, showing the WF and E_F_. (**b**) Schematic illustration of the vacuum level shift that can occur at the interface after contact between the donor and acceptor, showing different work functions (WF_1_ and WF_2_) The letters D and A denote Donor and Acceptor, respectively; VL is Vacuum Level; WF is Work Function; IP is Ionization Potential; EA is Electron Affinity; Γ′ is the photovoltaic gap; and E_F_ is the Fermi level [83].

Consider two materials with different work functions (WFs) or electrochemical potentials. When these materials come into contact, charge spontaneously redistributes at the interface. This charge redistribution occurs to achieve thermodynamic equilibrium, resulting in a unified Fermi level (E_F_) [82] across the interface. The process is known as interfacial charge transfer. Typically, electrons flow from the material with the higher E_F_ to the material with the lower E_F_. This charge transfer has two main consequences that together determine the interfacial electronic structure. Firstly, charge transfer or rearrangement occurs within a few atomic or molecular layers adjacent to the interface. This forms an interface dipole layer. This dipole causes a sudden step in the E_vac_ across the interface (Figure 7a). The step directly alters the relative energy level positions between the materials. Secondly, space charge regions form within the bulk materials on both sides of the interface. These regions form to neutralize the transferred charge. The net charge in these regions generates a built-in electric field. This field causes a gradual change in electrostatic potential within the bulk near the interface. This potential change leads to gradual bending (band bending) of all electronic energy bands (e.g., HOMO, LUMO, core levels). Figure 7b illustrates this band bending. Therefore, interface dipoles and band bending are two key physical mechanisms.

Charge transfer is common at organic/metal interfaces [84,85,86]. This commonly occurs due to the relative positions of the metal work function and relevant energy levels (e.g., LUMO) of the organic molecule, with electrons typically tending to transfer from the metal to the organic molecule. This charge transfer directly impacts the injection barriers and energy level alignment status at the interface. For example, in the Ni-TPP/Cu(100) system, studies confirmed that electron transfer from the copper substrate to Ni-TPP molecules leads to significant interfacial energy level bending [87]. Similarly, research by Okuyama et al. on the CuPc/Cu(100) interface also showed that charge transfer causes the LUMO level of CuPc to shift downward, promoting energy level alignment [88].

**Figure 8:**
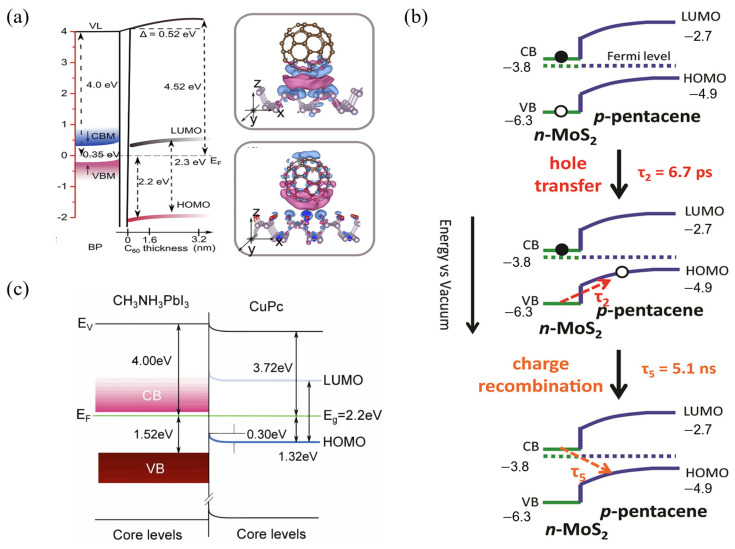
Energy Level Alignment and Band Bending in Organic Heterojunctions Driven by Interfacial Charge Transfer. (**a**) Energy level diagram of the C_60_/BP heterojunction and results of charge transfer and band bending in this heterojunction demonstrated by DFT calculations [89], copyright 2018, American Chemical Society. (**b**) Energy level diagram of the CH_3_NH_3_PbI_3_/CuPc heterojunction [90], copyright 2017, American Chemical Society. (**c**) Schematic diagram of exciton dynamics in the MoS_2_–pentacene heterojunction, illustrating the mechanisms of hole transfer and charge recombination [91].

Layered two-dimensional (2D) semiconductors often possess unique electronic structures and tunable band properties, making them ideal platforms for studying interfacial phenomena such as charge transfer. Black phosphorus (BP) is one such material that has garnered significant interest in these studies. Wang et al.’s research shows a significant difference in work function and electron affinity between C_60_ and BP [89]. C_60_ has a work function of 5.70 eV and an electron affinity of 2.68 eV, while BP has a work function of 4.00 eV and an electron affinity of 0.48 eV, as shown in Figure 8a. This significant difference in intrinsic energy levels provides an inherent thermodynamic drive for interfacial charge transfer. During C_60_/BP heterojunction construction, electrons spontaneously transfer from BP (lower WF) to C_60_ (higher work function) until Fermi level alignment is achieved. This electron transfer from BP to C_60_ forms a hole accumulation layer on the BP surface, inducing upward band bending. XPS experimentally confirms this band bending, with a 0.16 eV shift of the P 2p core level towards lower binding energy. Simultaneously, the C_60_ layer becomes n-type doped due to electron injection and builds up a significant interface dipole (0.36 eV) at the heterojunction interface, largely dominating the change in the heterojunction’s overall work function (0.52 eV). Finally, driven by interfacial charge transfer, a vacuum level shift of approximately 0.52 eV forms at the C_60_/BP heterojunction interface, achieving effective energy alignment between the C_60_ LUMO level and the BP conduction band minimum (CBM) level. This builds a built-in electric field within the heterojunction that favors efficient carrier separation and directional transport.

This charge-transfer-driven band bending is not limited to C_60_/BP but is also prevalent at other organic–inorganic interfaces involving emerging materials like van der Waals heterojunctions, influencing subsequent dynamic processes. Bettis Homan et al. investigated carrier dynamics in a p-n Type II van der Waals heterojunction composed of pentacene and monolayer MoS_2_ [90]. Figure 8b shows the energy band structure of the p-n heterojunction formed between MoS_2_ and pentacene. The CBM and VBM of MoS_2_ are located at −3.8 eV and −6.3 eV, respectively. The LUMO and HOMO of pentacene are positioned at −2.7 eV and −4.9 eV, respectively. Due to the Fermi level difference, electrons transfer from MoS_2_ to pentacene, forming a depletion region and a built-in electric field at the interface. This causes upward bending of pentacene’s HOMO and LUMO levels near the MoS_2_ interface, resulting in a band offset of approximately 1.1 eV. The band bending induced by charge transfer provides the energy driving force for hole transfer (τ_2_ = 6.7 ps) from MoS_2_ to pentacene after photoexcitation. Ultimately, this process creates long-lived charge-separated states (5.1 ns) at the interface.

Beyond the fundamental research systems mentioned above, interfacial charge transfer and band bending are also critically important for optimizing interfaces in perovskite solar cells. As shown in Figure 8b, Chen et al. further verified how interfacial charge transfer modulates band bending, using the CH_3_NH_3_PbI_3_/CuPc heterojunction system as a research object [91]. As shown in Figure 8c, the experimental results reveal a downward band bending of approximately 0.3 eV within the CuPc thin film, persisting significantly even at a thickness of 36 nm. The energy level alignment facilitates hole transfer, which is identified as a potential driver of the band bending. Energy level analysis demonstrates that the HOMO level of CuPc lies ~1.32 eV below the Fermi level when the film reaches 36 nm thickness. This energy configuration promotes hole transfer from CH_3_NH_3_PbI_3_ to CuPc, inducing localized electrostatic potential variations that generate the observed downward band bending. Spiro-OMeTAD exhibits downward band bending (about 0.3 eV) at its interface with perovskite due to interfacial charge transfer [92]. UPS measurements by Schulz et al. further reveal that this charge-transfer-induced band bending persists within the Spiro-OMeTAD layer without attenuation even at the maximum measured thickness (8 nm), suggesting the actual bending magnitude may be significantly larger. In contrast, Chen et al. observed significantly smaller band bending (0.1–0.2 eV) induced by interfacial charge transfer at the CuPc/CH_3_NH_3_PbI_3_ interface with an 8 nm CuPc thickness. The authors conclude that CuPc outperforms Spiro-OMeTAD as a hole transport material in perovskite solar cells in terms of charge-transfer-induced band bending. The reduced band bending in CuPc implies less obstruction to hole transport at the interface, which is critical for efficient hole extraction.

#### 3.1.3. Interface Band Bending Caused by Interfacial Chemical Reactions

In addition to physical charge transfer driven by initial energy level mismatch, interfacial chemical reactions are another crucial factor regulating electronic structure. Chemical reactions can generate electronic states at the interface within the semiconductor band gap. These are known as interface states. These interface states may originate from dangling bonds, reaction-induced defects, or the electronic structure of newly formed chemical species at the interface. The density and energy distribution of interface states are key factors determining band bending’s degree and nature. When the interface state density is sufficiently high, these states can pin the Fermi level at the interface. The Fermi level becomes fixed at a specific energy within the semiconductor band gap. This pinning is largely independent of the contacting material’s work function. This Fermi level pinning requires significant band bending in the semiconductor. This bending aligns the bulk Fermi level with the pinned surface Fermi level. Therefore, interface states generated by chemical reactions can dominate band bending behavior. Identifying and understanding the specific interfacial chemical processes is crucial for predicting and controlling the final interfacial electronic structure. Different types of reactions affect band bending through distinct mechanisms.

Direct chemical bonding between metals and organic molecules upon contact can significantly alter the interfacial charge distribution and electronic states. The specific effects depend strongly on the metal’s reactivity. When metallic lithium (Li) is deposited on the poly(3-hexylthiophene) (P3HT) surface, Li atoms have low electronegativity. When deposited, these atoms do not merely physisorb on the P3HT surface. Instead, they chemically react with the polymer. The reaction involves sulfur (S) and carbon (C) atoms within P3HT’s thiophene rings. This process forms Li_2_S and Li-C complexes. In the reaction, Li atoms lose outer-shell electrons, becoming Li^+^ ions, and transfer these electrons to the P3HT polymer’s molecular orbitals. This electron injection from the metal to the semiconductor polymer leads to net negative charge accumulation in the P3HT region near the interface. According to semiconductor physics theory, this space charge region formation near the interface generates a built-in electric field pointing into the polymer, altering the electrostatic potential in that region. Macroscopically, this electrostatic potential change manifests as the overall downward bending of P3HT’s electronic energy bands (including HOMO, LUMO, and E_vac_ relative to the Fermi level, reducing the barrier for electron injection from Li to P3HT. The band bending observed here results primarily from two effects. First is the n-type doping effect caused by electron injection from Li. Second is the formation of a space charge region.

Compared to reactive lithium, noble metals like silver interact more weakly with P3HT. However, they still affect the interfacial electronic structure through chemical reactions. Research has examined the Ag/P3HT interface, especially at low coverage [93]. XPS confirmed a chemical interaction between P3HT sulfur atoms and the Ag substrate. This interaction forms S-Ag bonds similar to those in metal sulfides. It was mainly confined to the region immediately adjacent to the interface. This chemical interaction is considered the primary reason for changes in the interfacial electronic structure. Experiments observed a significant decrease in the P3HT vacuum level relative to Ag. This formed an interface dipole of approximately 0.36 eV. The dipole pointed toward P3HT. Simultaneously, P3HT core levels and valence band edge shifted toward higher binding energy. This shift was approximately 0.2 eV relative to the Fermi level. It indicated downward band bending within the P3HT bulk. The authors attributed the vacuum level decrease and electronic structure changes to electron transfer from P3HT to Ag. They also explicitly stated that interface dipole formation closely relates to the S-Ag chemical interaction. Therefore, at this interface, chemical interactions dominate charge redistribution. This leads to interface dipole formation and band bending. Thus, reactive metals primarily drive band bending through n-type doping and space charge region formation. In contrast, inert metals induce smaller magnitude band bending. This occurs primarily through localized interface dipoles formed by chemical bond generation.

**Figure 9:**
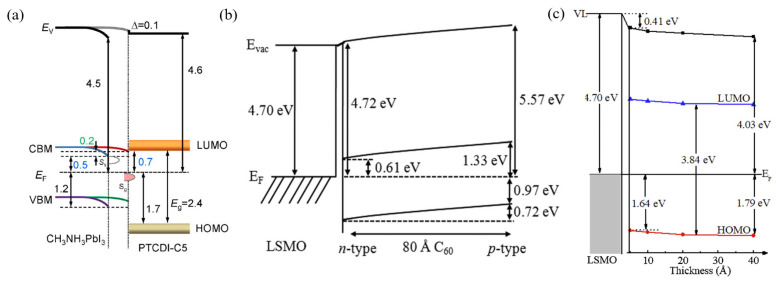
Influence of Interfacial Chemical Reactions on Energy Level Alignment and Band Bending in Organic Heterojunctions. (**a**) Energy level diagram of the PTCDI-C_5_/CH_3_NH_3_PbI_3_ heterojunction, illustrating reaction-induced changes in perovskite surface band bending [94]. (**b**) Energy level diagram of the C_60_/LSMO heterojunction, revealing oxygen diffusion-driven band bending and doping type transition in C_60_ [95]. (**c**) Energy level diagram of the C8-BTBT/LSMO heterojunction, showing the energy difference between the LSMO Fermi level and the C8-BTBT HOMO [96].

Chemical interactions also frequently occur at interfaces between small organic molecules and inorganic materials (e.g., perovskites). These reactions can alter the inorganic material’s surface states or doping status. They can also form new interface layers. These changes consequently modulate the band structure. In perovskite solar cells, the chemical interaction between PTCDI-C_5_ molecules and the CH_3_NH_3_PbI_3_ perovskite surface is one such instance [72]. These interactions altered the perovskite’s surface band bending. They also adjusted energy level alignment via charge transfer. Consequently, these changes affected charge extraction in the device. Zhang et al. performed an interface study of PTCDI-C5 with CH_3_NH_3_PbI_3_ perovskite [94]. Figure 9a provides an intuitive illustration of this process. Pristine CH_3_NH_3_PbI_3_ exhibits downward band bending of ~0.2 eV (inferred from work function and Fermi level positions). After PTCDI-C_5_ deposition and chemical reaction, this downward bending is significantly reduced. Specifically, the CH_3_NH_3_PbI_3_ work function decreases from 4.5 eV to 4.4 eV, consistent with electron transfer from PTCDI-C_5_ to the CH_3_NH_3_PbI_3_. Although the LUMO of PTCDI-C5 is nearly aligned with the CBM of the perovskite, which is favorable for electron extraction, a large energy difference (0.7 eV) exists between its HOMO and the VBM of the perovskite, which might hinder hole transport. This modulation of interface energy levels is considered to be achieved by chemical reactions that alter the perovskite’s surface states and surface potential.

Chemical reactions at organic–inorganic interfaces can also alter existing band bending. They do this by affecting surface states and carrier concentration in the inorganic semiconductor. Interfacial reactions involving atomic diffusion across the interface are of particular interest. In previous research, Xie et al. explicitly revealed that oxygen diffusion, a core interfacial chemical reaction, drives band bending at the C_60_/LSMO interface [95]. As shown in Figure 9b, as C_60_ thickness increases, its work function increases from 4.72 eV to 5.57 eV, the HOMO level moves approximately 0.72 eV closer to the Fermi level, and upward band bending of 0.72 eV occurs, accompanied by C_60_ transitioning from n-type to p-type. These electronic structure changes occur synchronously with oxygen diffusion (O/Mn ratio increases from 3.11 to 5.05, Mn^3+^/Mn^4+^ ratio increases from 2.35 to 3.44). Oxygen atom diffusion from LSMO to C_60_ is driven by differences in oxygen vacancy concentration or the tendency to form more stable chemical bonds. Furthermore, these diffusing atomic species can participate in or initiate subsequent chemical reactions. This makes the interface evolution more complex. Xie et al. further verified the generality of interfacial chemical reactions in the C8-BTBT/LSMO system [96]. As shown in Figure 9c, at the C8-BTBT/LSMO interface, the LSMO Fermi level is above the C8-BTBT HOMO, indicating a significant hole injection barrier. As C8-BTBT film thickness increased from 0 Å to 120 Å, the O/Mn atomic ratio significantly increased from approximately 3.1 to nearly 4.8, directly confirming oxygen atom diffusion from LSMO to the C8-BTBT film. Under ultraviolet irradiation during UPS measurements, the diffused oxygen participated in a photochemical interaction with C8-BTBT molecules, generating distinct chemical species. These species are identified in the XPS spectra through characteristic binding energies at 286.18 eV (C 1s) and 163.73 eV (S 2p), corresponding to oxidized sulfur–carbon moieties. These chemical changes demonstrate the modulation of energy level alignment and interfacial electronic properties by interfacial chemical reactions.

In short, interfacial chemical reactions, through mechanisms like direct bonding or atomic diffusion, profoundly alter the electronic structure and band bending at organic interfaces. These chemical effects are crucial for device performance and stability. Understanding these processes thus motivates the use of interface engineering techniques, such as the interlayers and template layers discussed in the following section (Section 3.2), to control and optimize interfacial properties.

### 3.2. Interface Engineering

Building upon the thorough analysis of the interface band bending mechanism elaborated in the preceding content, interface engineering, an integral and pivotal technology within the realm of organic electronics, serves as a critical conduit for effecting substantial improvements in device performance. At its core, it involves meticulous and precise control over the microscopic features of the interfaces of organic semiconductor materials. Through the regulation of molecular orientation, energy level configurations, and charge carrier transport processes, the objective of optimizing device performance can be ultimately realized.

#### 3.2.1. Interlayer Control

Inserting functional thin films at interfaces is a common and effective strategy. These films are known as interlayers which can modulate interfacial electronic structure and optimize device performance. It is typically achieved by selecting materials with suitable work functions for energy level matching. Molybdenum trioxide (MoO_3_) is a transition metal oxide with a high work function and is widely used as a hole injection layer (HIL) or electron blocking layer (EBL) [97,98,99]. It is widely used in various devices, including OLEDs [100,101,102], OTFTs [103], and OSCs [97,98,99]. The WF, IP, and electron affinity (EA) of MoO_3_ are 6.7 eV, 9.4 eV, and 6.2 eV, respectively [104,105,106]. Its high work function helps reduce the barrier between the organic layer and the anode electrode. Studies have shown that MoO_3_ can induce significant interfacial effects upon contact with organic semiconductors. For example, it can lead to strong p-type doping of C_60_. It also causes band bending extending up to hundreds of angstroms at the C_60_/MoO_x_ interface [107]. At the α-NPD/MoO_3_ interface, MoO_3_ effectively enhances hole injection and extracts electrons from the α-NPD [108]. Similarly, inserting a MoO_3_ layer just a few nanometers thick between a ferromagnetic metal electrode and an organic semiconductor also effectively reduces the hole injection barrier [99,107,109]. Chen et al. find that MoO_3_ interlayers can be used to construct efficient charge generation layers (CGLs) [18]. Inserting a MoO_3_ buffer layer in a C_60_/pentacene system reduced the injection barrier and significantly improved tandem OLED power efficiency.

**Figure 10:**
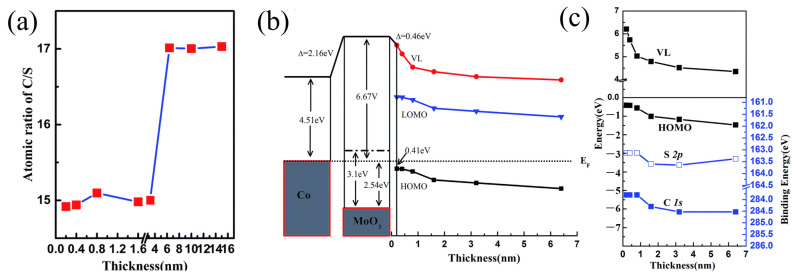
Modulation of Electronic Structure and Chemical Reactions at the C8-BTBT/Co (100) Interface by a MoO_3_ Buffer Layer. (**a**) The measured atomic ratio of C/S (red squares) as a function of C8-BTBT thickness. (**b**) Schematic energy diagram for C8-BTBT/MoO_3_/Co (100). (**c**) Evolutions of the E_vac_, HOMO, S 2p, and C 1s as the C8-BTBT thickness increases [23].

Interlayers can also passivate interfaces and block detrimental chemical reactions. These actions improve device stability. As another example involving MoO_3_, Zhu et al. investigated interface electronic structure at the C8-BTBT/Co(100) interface by introducing MoO_3_ [23]. It shows that a MoO_3_ buffer layer can effectively suppress desulfurization at the C8-BTBT/Co interface. As shown in Figure 10a, at the C8-BTBT/MoO_3_/Co interface, the C/S atomic ratio remains close to the theoretical value of 15:1. Meanwhile, the S 2p and C 1s peak positions shift synchronously toward lower binding energy. It indicates that interfacial chemical bonds remain intact, inhibiting the desulfurization reaction. Furthermore, the energy band bends downward with increasing C8-BTBT thickness, as shown in Figure 10b. This energy level alignment, specifically the MoO_3_ CBM being lower than the C8-BTBT HOMO (6.2 eV vs. 5.8 eV), facilitated electron transfer from the C8-BTBT HOMO to the MoO_3_ CBM, significantly reducing the hole injection barrier from 1.41 eV (at the direct C8-BTBT/Co interface) to 0.97 eV (at the C8-BTBT/MoO_3_ interface). As shown in Figure 10c, the E_vac_ and HOMO level continuously shifted downward with increasing C8-BTBT thickness, reflecting band bending in C8-BTBT.

**Figure 11:**
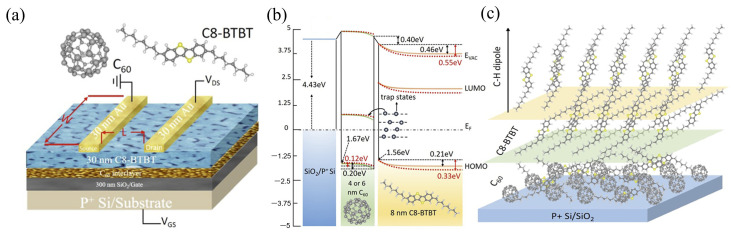
Energy Level Alignment and Molecular Packing at the C8-BTBT/C_60_/SiO_2_ Interface. (**a**) Schematic of the bottom-gate, top-contact OFET structure, with insets showing C_60_ and C8-BTBT molecular structures. (**b**) Energy level alignment diagram of the C8-BTBT/C_60_/SiO_2_ interface with 4 nm (orange solid line) and 6 nm (red dashed line) C_60_ interlayers, showing the E_vac_ (**c**) C8-BTBT molecular packing mode on C_60_/SiO_2_ [112].

Besides modulating energy levels, interlayers can act as specialized charge transport layers or buffer layers, which can improve carrier transport or interfacial contact. Fullerene C_60_ is often used as an electron transport layer (ETL) or electrode buffer layer [110]. This is due to its good electron transport properties. Yao et al. studied pentacene organic phototransistors (OPTs) [111]. By introducing a C_60_ buffer layer between the source and drain electrodes the related devices showed higher performance under illumination compared to traditional single-layer pentacene OPTs. The maximum photoresponsivity reached 4.27 A W^−1^ which is six times higher than that of single-layer devices. Meanwhile, the C_60_-modified devices achieved a lower threshold voltage and higher field-effect mobility [111]. This improvement is attributed to the C_60_ layer effectively reducing the hole injection barrier. The C_60_ layer also facilitates photogenerated exciton dissociation via the pentacene/C_60_ heterojunction. Zhao et al. showed that a C_60_ interlayer significantly modulated the interfacial electronic structure of C8-BTBT films [112]. Figure 11a illustrates the OFET device structure, a bottom-gate top-contact configuration. The inserted C_60_ nanolayer is located between the C8-BTBT film and the SiO_2_ gate insulator. C_60_, acting as a strong acceptor with its LUMO level within the C8-BTBT band gap, can trap electrons from C8-BTBT trap states, reducing trap state density; this is expected to lower the OFET’s threshold voltage. However, the C_60_ nanolayer can also increase disorder at the C8-BTBT interface, potentially reducing carrier mobility. As shown in Figure 11b, an interface dipole of 0.40 eV was observed for a 4 nm C_60_ interlayer. This is attributed to the higher work function of C_60_ (4.85 eV) compared to C8-BTBT (4.25 eV), leading to electron transfer from C8-BTBT to C_60_. Significant band bending, primarily caused by the built-in electric field, was observed near the interface in both the C8-BTBT and C_60_ layers. Notably, the 6 nm C_60_ interlayer induced more pronounced band bending in C8-BTBT and the shift value is approximately 0.21 eV (4 nm C_60_) and 0.33 eV (6 nm C_60_), respectively. The larger band bending implies a higher hole accumulation concentration, potentially explaining the improved device performance observed with the 6 nm C_60_ interlayer. As shown in Figure 11c, a C_60_ layer was introduced. As C8-BTBT thickness increased, the molecules gradually adopted a highly ordered upright arrangement.

Furthermore, some molecules with special optoelectronic functions are also used as interlayers. Pentacene is an organic semiconductor with unique singlet fission characteristics. Singlet fission is the process where a high-energy singlet exciton splits into two low-energy triplet excitons. In solar cells, this process effectively converts one high-energy photon into two electron–hole pairs. This could potentially increase device quantum efficiency to over 100%. Incorporating a monolayer pentacene interlayer in PbSe and silicon-based solar cells improved device performance [113]. Specifically, it can improve the carrier separation efficiency and overall solar cell efficiency. Despite pentacene having significant potential for singlet fission, its inherent air instability and low solubility limit widespread use in practical devices [114,115,116]. Researchers are actively developing pentacene derivatives to overcome these limitations. Among these, TIPS-PEN has attracted considerable attention. This attention is due to its excellent air stability and solution processability. Inserting a TIPS-PEN interlayer has been shown to significantly improve photodetector performance [117]. It also improves the detection rate for specific wavelengths, such as green light.

In summary, interlayer materials and structures can be carefully selected and designed. This allows various interfacial problems to be addressed in a targeted manner. These problems include energy level mismatch, inefficient carrier injection/transport, and chemical instability. This makes interlayer design an indispensable part of interface engineering.

#### 3.2.2. Template Layer Control

Researchers have incorporated a template layer composed of one or two molecular layers between the insulating layer and the organic semiconductor layer. This approach aims to optimize various aspects of organic semiconductor films, including their morphology, molecular orientation, and interfacial properties. Through this molecular template layer mediated growth method, the performance of organic semiconductor field-effect transistors can be effectively improved [118,119,120,121,122]. In the phthalocyanine metal films that undergo weak epitaxial growth on p-6P, the organic field-effect transistors fabricated using VOPc films demonstrate the highest mobility, with a value surpassing 1 cm^2^/Vs [123,124]. Yan et al. successfully grew high-quality and highly ordered CuPc films on a BP2T template layer. The resulting heterojunction led to a three orders of magnitude increase in the electrical conductivity of the CuPc films when compared to CuPc films without a template layer [125].

**Figure 12:**
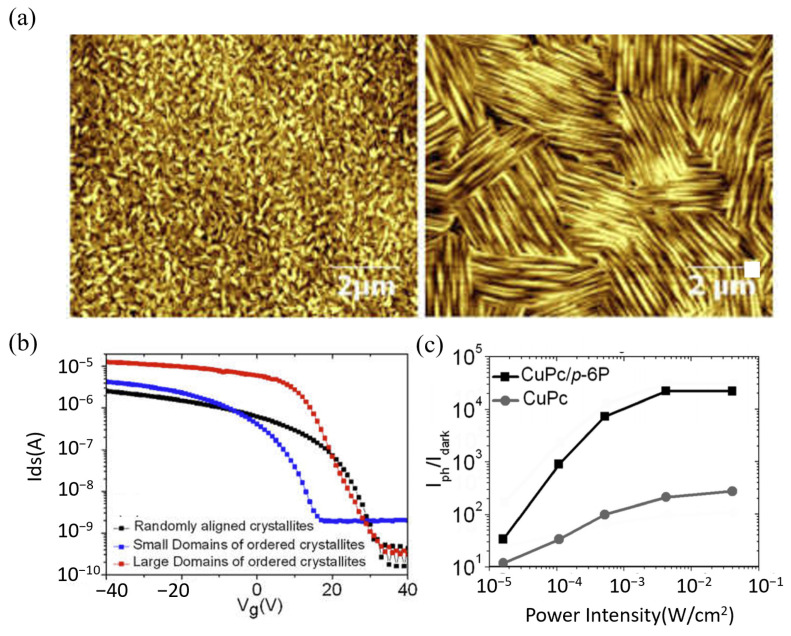
Influence of p-6P molecular template on CuPc thin film morphology and performance of resulting organic field-effect transistors and phototransistors. (**a**) AFM images comparing CuPc thin film morphologies. Left: Randomly arranged CuPc grains grown without a p-6P template. Right: Large, ordered CuPc crystalline domains grown on a p-6P template. (**b**) Transfer characteristic curves of OFETs based on the different CuPc films. The red curve corresponds to the device with large, ordered domains (template-grown) and exhibits the highest field-effect mobility [126]. (**c**) Photocurrent/dark current ratio versus light power for phototransistors. Devices are based on CuPc/p-6P heterojunctions or pure CuPc films. The black curve (CuPc/p-6P heterojunction device) shows a higher ratio at the same light power [24].

In 2015, Qian et al. used a p-6P molecular template to induce the growth of CuPc thin film [126]. The template promoted larger single-crystal domains by inducing specific molecular alignment. It can significantly reduce the grain boundaries, which impede charge transport (Figure 12a). OFETs were fabricated using these large-domain CuPc films. As shown in Figure 12b, these devices showed improved performance compared to OFETs with non-templated, randomly oriented CuPc films. Field-effect mobility increased from 7.5 × 10^−3^ cm^2^/Vs) to 0.18 cm^2^/Vs). The on/off ratio improved from 5 × 10^3^ to 4.2 × 10^4^. Device stability was also significantly enhanced. In 2017, Qian et al. used a p-6P molecular template to help grow high-quality CuPc thin films [24]. As shown in Figure 12c, the CuPc/p-6P heterojunction structure significantly enhanced phototransistor photoresponse. Under 365 nm ultraviolet illumination, the CuPc/p-6P heterojunction device showed a high photoresponsivity of 4.3 × 10^2^ A W^−1^. It is much higher than that of pure CuPc devices (7.3 A W^−1^). It can be attributed to the p-6P layer enhancing UV light absorption. It also separated and transported photogenerated carriers more effectively at the heterojunction interface. This resulted in high-performance photodetection. Therefore, the p-6P molecular template is crucial for significantly improving organic device performance by modulating CuPc thin film microstructure. This modulation enhances charge transport efficiency and device stability. It also boosts light absorption and photogenerated carrier generation.

Besides p-6P, various materials, including PTCDA, HATCN, and self-assembled monolayers (SAMs), are also commonly used as template layers for organic semiconductors. PTCDA primarily influences molecular orientation through its planar structure and π–π interactions with organic semiconductors. HATCN, with its strong electron-accepting properties and high work function, is often used to tune energy level alignment and promote charge injection. SAMs provide customized interfaces for organic semiconductor growth by flexibly controlling the surface properties of the substrate. These materials each have unique characteristics and play important roles in interface engineering of organic electronic devices. Thin PTCDA template layers significantly influence the crystal orientation of subsequently deposited CuPc thin films. While CuPc typically adopts an upright (100)-α phase molecular configuration, a PTCDA template layer induces a nearly flat-lying CuPc orientation. This reorientation improves π-orbital overlap between CuPc molecules, enhancing exciton diffusion and charge transport [127]. In organic photovoltaic devices, a 10 Å PTCDA interlayer increased the short-circuit current density (J_sc_) to 4.06 mA cm^−2^, nearly 60% higher than that of devices without a PTCDA interlayer (2.56 mA cm^−2^) [128]. A truxenone monolayer on Cu(111) has been successfully used as a template for growing highly ordered monolayer and bilayer C_60_ thin films. STM and low-energy electron diffraction (LEED) results have shown that C_60_ forms a commensurate 8 × 8 superstructure on the truxenone/Cu(111) template, indicating a high degree of induced structural order [129,130]. In recent years, two-dimensional materials have also become highly attractive template materials due to their atomically flat surfaces and unique electronic properties. For example, graphene is an effective substrate for growing ultrathin C8-BTBT films. Continuous C8-BTBT growth on graphene enables studying photoresponse as a function of the number of C8-BTBT layers in phototransistors. Even a single C8-BTBT layer yields phototransistors with a high photoresponsivity of up to 1.57 × 10^4^ A W^−1^ and a fast response time as short as 25 ms [131]. In few-layer C8-BTBT/graphene phototransistors, interfacial charge transfer efficiency reached 41%, and photoconductive gain exceeded 10. The initial growth mode and final morphology of C8-BTBT films are also influenced by other substrates, such as single-crystal Al_2_O_3_ (C-sapphire) [132,133,134]. Studies show that C8-BTBT tends to lie flat on graphene, C-sapphire, and boron nitride (BN) at low surface coverage due to strong π–π interactions, while adopting an upright growth mode at higher coverage. The results highlight the crucial role of substrates in guiding C8-BTBT molecule self-assembly [132,133,134].

## 4. Conclusions

Energy band bending is a common phenomenon observed at organic semiconductor interfaces. They determine the performance of organic electronic devices. The final energy level structure depends jointly on material electronic properties, microstructure, and interface interactions. As reviewed in this paper, the energy band bending induced by molecular orientation, interfacial charge transfer, and interfacial chemical reactions has been discussed in detail. Molecular orientation affects carrier transport and surface work function/ionization energy. Interfacial charge transfer drives dipole formation and band bending. Interfacial chemical reactions act through interface states or doping effects. Understanding and effectively controlling these factors are crucial for optimizing charge injection, extraction, transport, and recombination.

Over the past decades, researchers developed various interface engineering strategies. Common strategies include interlayer insertion and template layers. Interlayers can tune energy levels or provide surface passivation, among other functions. Template layers control morphology and orientation. Significant progress has been made in improving device performance using these strategies. However, the field still faces severe challenges. These include difficult-to-overcome Fermi level pinning effects and persistently high contact resistance. Insufficient interface stability and a lack of reliable predictive models are also major issues. These problems limit further enhancement and widespread application of organic electronic device performance.
